# Mapping Knowledge Structure and Themes Trends of Osteoporosis in Rheumatoid Arthritis: A Bibliometric Analysis

**DOI:** 10.3389/fmed.2021.787228

**Published:** 2021-11-23

**Authors:** Haiyang Wu, Kunming Cheng, Qiang Guo, Weiguang Yang, Linjian Tong, Yulin Wang, Zhiming Sun

**Affiliations:** ^1^Department of Clinical Medicine, Graduate School of Tianjin Medical University, Tianjin, China; ^2^Department of Intensive Care Unit, The Second Affiliated Hospital of Zhengzhou University, Zhengzhou, China; ^3^Department of Orthopaedic Surgery, Baodi Clinical College of Tianjin Medical University, Tianjin, China; ^4^Department of Orthopaedic Surgery, Tianjin Huanhu Hospital, Tianjin, China

**Keywords:** rheumatoid arthritis, osteoporosis, bibliometrics, CiteSpace, VOSviewer

## Abstract

**Background:** Rheumatoid arthritis is a chronic disabling disease characterized by chronic inflammation, articular cartilage destruction, and reduced bone mass. Multiple studies have revealed that the development of osteoporosis in rheumatoid arthritis (RA; ORA) patients could be led to a reduced quality of life and increased healthcare costs. Nevertheless, no attempt has been made to analyze the field of ORA research with the bibliometric method. This study aimed to provide a comprehensive overview of the knowledge structure and theme trends in the field of ORA research from a bibliometric perspective.

**Methods:** Articles and reviews regarding ORA from 1998 to 2021 were identified from the Web of Science database. An online bibliometric platform, CiteSpace, and VOSviewer software were used to generate visualization knowledge maps including co-authorship, co-citation, and co-occurrence analysis. SPSS, R, and Microsoft Excel software were used to conduct curve fitting and correlation analysis, and to analyze quantitative indicators, such as publication and citation counts, *h*-index, and journal citation reports.

**Results:** A total of 1,081 papers with 28,473 citations were identified. Publications were mainly concentrated in North America, Western Europe, and Eastern Asia. Economic strength is an important factor affecting scientific output. The United States contributed the most publications (213) with the highest *h*-index value (46) as of September 14, 2021. Diakonhjemmet Hospital and professor Haugeberg G were the most prolific institution and influential authors, respectively. *Journal of Rheumatology* was the most productive journal concerning ORA research. According to the burst references, “anti-citrullinated protein antibodies” and “preventing joint destruction” have been recognized as the hot research issues in the domain. The keywords co-occurrence analysis identified “teriparatide,” “interleukin-6,” “Wnt,” and “vertebral fractures” as the important future research directions.

**Conclusion:** This was the first bibliometric study comprehensively summarizing the trends and development of ORA research. Our findings could offer practical sources for scholars to understand the key information in this field, and identify the potential research frontiers and hot directions in the near future.

## Introduction

Rheumatoid arthritis is an autoimmune disorder with a nearly 1% prevalence in the global population and is generally associated with significant morbidity and mortality (1). Chronic inflammation, a hallmark feature of rheumatoid arthritis (RA) disease, causes articular cartilage destruction and bone erosion, which subsequently leads to generalized osteoporosis with reduced bone mass (2, 3). Development of osteoporosis in RA (hereafter ORA) patients results in a further reduced quality of life and increased healthcare costs. Apart from that, abundant studies have demonstrated that the incidence of osteoporotic fractures in patients with RA is higher than in the matched non-RA population (4–7). It was estimated that the occurrence of femoral neck fractures and vertebral compression fractures in these patients was increased 2-fold than a healthy population of the same age (6). Data from the National Data Bank for Rheumatic Diseases in the USA suggested that osteoporotic fracture was the third leading cause of mortality in RA patients (1). Therefore, the updated American College of Rheumatology (ACR) guidelines and recommendations for RA treatment have stressed the prevention and control of ORA patients.

In view of the aspects described above. ORA has received increasing attention from scholars. However, to date, relatively little is known about the exact pathogenetic mechanisms that determine the severity of bone loss (8). In addition, there are still some controversies derived from ORA, such as the fracture risk assessment and risk factors, the effectiveness of disease-modifying antirheumatic drugs (DMARDs) on preventing bone loss, time-window for fracture prevention, and the optimal anti-osteoporotic protocols, and so on (9–12). Motivated by these concerns, a considerable amount of research related to ORA was published and this topic has gained increasing attention among scholars. Nevertheless, the rapidly increasing number of publications makes it more and more difficult for researchers to keep up with the latest findings, even inside their domain of expertise. Although several systematic reviews and meta-analyses surrounding this topic could offer innovations and basic information to researchers, these summative reviews merely focus on a unique perspective of ORA research, while some meaningful information such as the numerical growth trend, the contributions of countries, institutions, and authors, prediction of future research hotspots are not included (10, 13). Some studies pointed out that early-career researchers could benefit from the overview analysis of knowledge structure and current hotspots in a certain field (14, 15). Given this, bibliometric analysis has become an increasingly popular approach to acquiring the above-mentioned parameters.

Bibliometrics is a feasible method to analyze the scientific production quantitatively and qualitatively and the current status of a given research field. With the development of information technologies, the information visualization of bibliometrics has been achieved and several freeware bibliometric tools including CiteSpace (16), VoSviewer (17), R-bibliometrix (18), and HistCite (19) have been widely used medical fields such as orthopedics (20), neurology (21), oncology (22, 23), and rheumatology (24). Taking RA and osteoporosis as examples, Schöffel et al. (24), have performed the first bibliometric analysis of 78,128 documents regarding RA during the period 1901–2007. And their analysis has revealed the most prolific authors, institutions, and journals dealing with the topic. Wang et al. conducted a bibliometric study based on the WoS database to explore the publication status and research hotspots in the field of RA-related depression (25). Additionally, several scholars also investigated the publications on osteoporosis by using bibliometric methods and mapped the overall knowledge structures of the field (26, 27). However, as far as we know, although there had been several bibliometric studies on RA or osteoporosis, no attempt has been made to analyze the field of ORA to date. In order to fulfill this knowledge gap, this study aimed to make an overall analysis of scientific publications on ORA research from 1998 to 2021, thus identifying the main contributors and current research status, as well as presenting prospects for future development of this field.

## Materials and Methods

### Data Acquisition and Search Strategy

**Figure 1:**
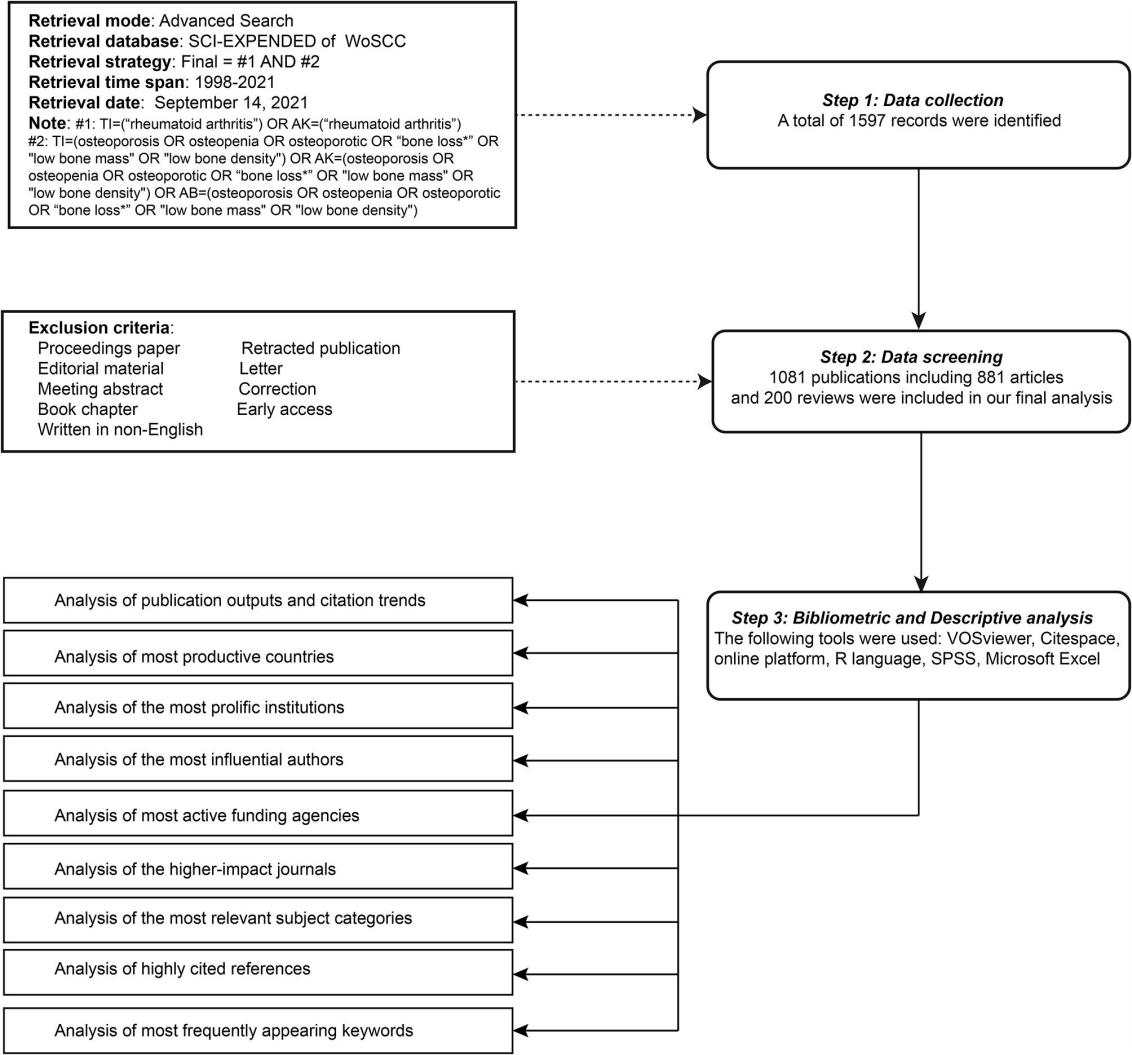
Flowchart of literature selection and data analysis.

Web of Science (WoS, Clarivate Analytics, Philadelphia, PA, USA), which contains more than 12,000 international academic journals, is one of the most comprehensive and authoritative database platforms to obtain global academic information (28). Apart from the general literature search, it also possesses an important function of citation index searching, which is helpful for assessing the academic performance of literature in a specific field. In our study, all the documents were retrieved and downloaded from the Science Citation Index Expanded (SCI-Expanded, 1998-present) of the WoS Core Collection (WoSCC) database on September 14, 2021, to avoid bias due to daily updates of the database. The search formula was set with reference to previous studies. Among these, as RA was the main research subject of this study, in order to achieve more precise results, terms related to “rheumatoid arthritis” were searched based on the titles (TI) and author keywords (AK). While terms related to “osteoporosis” were searched based on the TI, abstracts (AB), and AK. The specific search formula was as follows (Figure 1): #1: TI=(“rheumatoid arthritis”) OR AK = (“rheumatoid arthritis”); #2: TI = (osteoporosis OR osteopenia OR osteoporotic OR “bone loss^*^” OR “low bone mass” OR “low bone density”) OR AK = (osteoporosis OR osteopenia OR osteoporotic OR “bone loss^*^” OR “low bone mass” OR “low bone density”) OR AB = (osteoporosis OR osteopenia OR osteoporotic OR “bone loss^*^” OR “low bone mass” OR “low bone density”); final dataset: #1 AND #2. A total of 1,597 publications were retrieved, of which 516 invalid records including proceedings paper, editorial material, correction, meeting abstract, letter, early access, retracted publication, and non-English works of literature were excluded. Ultimately, 1,081 valid documents were obtained as the final dataset and exported in the form of “full record and cited references” for further analysis. Afterward, the plain text files were renamed for further analysis as CiteSpace software can only recognize files named with the specified name of “download*.txt

### Data Extraction

The final dataset was first imported into CiteSpace software (Chaomei Chen, Drexel University, USA) to remove duplicates. Then two independent researchers (WHY and CKM) performed the data extraction to ensure the accuracy and reliability of the results. Any disagreements among the two investigators were discussed until consensus was reached. The extracted data included publication counts, citation times, countries, institutions, authors, funding agencies, subject categories, journals, highly-cited articles, and keywords. By using the function of “Create Citation Report” in WoSCC, the Hirsch index (*h*-index), and Average Citations per Item (ACI) of counties, institutions, and authors were acquired. The journal information including impact factor (JIF) and Quartile in category (Q1, Q2, Q3, and Q4) was obtained from the 2020 Journal Citation Reports (Clarivate Analytics, Philadelphia, PA, USA). Moreover, in consideration of the differences in economic and demographic conditions in different countries, several ratio indices including the number of papers per million people, and several papers per trillion Gross Domestic Product (GDP) was introduced (28).

### Data Analysis

Statistical analysis was performed using SPSS (IBM SPSS Statistics 21, Inc., Chicago, IL, USA), R software (v3.6.3., R Foundation, Vienna, Austria), and Microsoft Excel 2019 (Microsoft Corporation, Redmond, WA, USA). Categorical data were expressed as count (percentage). The growth rate of publications over time was calculated with the following formula reported by Guo et al. (29). Growth rate = [(number of documents in the last year ÷ number of documents in the first year)^1/(last year−*first year*)^ – 1] × 100. The strength of correlation between continuous variables was assessed using Pearson's correlation coefficient. The strength of correlation coefficients was interpreted as follows: 0.00–0.25 as little if any correlation, 0.26–0.49 as low correlation, 0.5–0.69 as moderate correlation, 0.7–0.89 as high correlation, 0.9–1 as very high correlation. Correlations were considered statistically significant when the *p*-value was < 0.05.

Bibliometric and visualization analyses were conducted by three bibliometric tools. CiteSpace, free Java-based software developed by Chen (16), is one of the most popular bibliometric tools for visualizing and analyzing the scientific literature and is often used to ascertain the knowledge structure, distribution, as well as evolution of a given field. In our study, CiteSpace was utilized to (a) perform a cooperation analysis of institutions; (b) analyze the co-citation relationship of authors; (c) conduct a dual-map overlay of scientific journals; (d) perform a co-citation analysis of references; (e) identify the top 25 references with the strongest citation bursts. In the network maps, the nodes represent various items such as institutions, authors, and references. The node size and color rings indicate the number of these items and different years, respectively. The lines between the nodes reflect the cooperation or co-citation relationships of items (28, 30).

VOSviewer, another bibliometric software developed by Professor van Eck and Waltman (31), has text mining capabilities to extract important parameters from a large pool of scientific publications for construction and visualization of co-authorship, co-citation, and co-occurrence network (32). In this research, this software was mainly applied to conduct visualization networks including institution co-authorship analysis, author co-authorship analysis, journal co-citation analysis, and keywords co-occurrence analysis. In addition, VOS viewer is able to provide three types of network maps, including the network visualization map, the overlay visualization map, and the density visualization map. For detailed descriptions of these maps, one can find the software manual at https://www.vosviewer.com/documentation.

Moreover, an online bibliometric platform (https://bibliometric.com/) was also applied to conduct the collaboration analysis of countries and annual publication trend analysis.

## Results and Discussion

### Publication Outputs and Citation Trends

**Figure 2:**
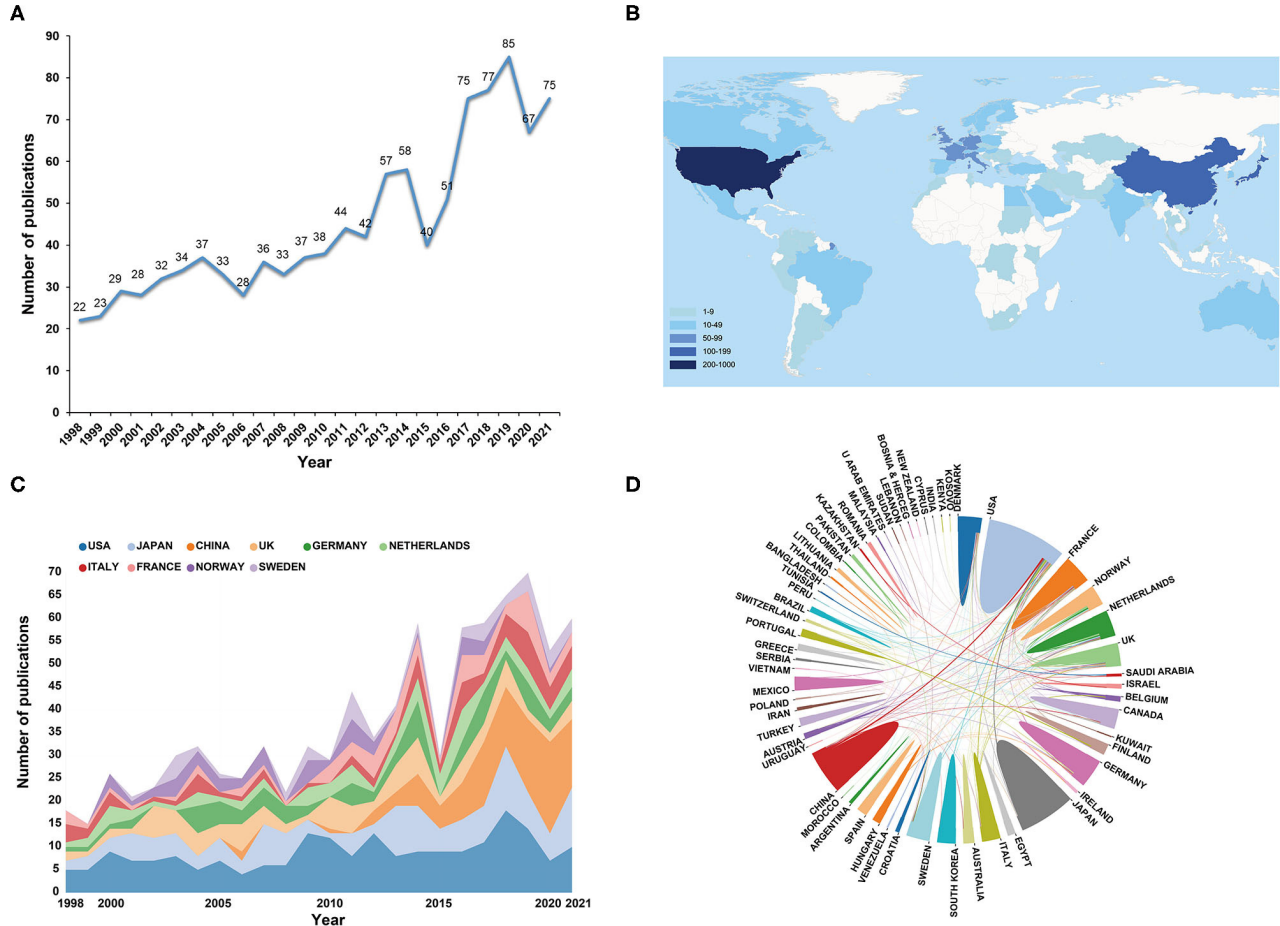
**(A)** The annual number of publications regarding osteoporosis in RA research from 1998 to 2021. **(B)** A world map depicting the contribution of each country based on publication counts. **(C)** The annual number of publications in the top 10 most productive countries from 1998 to 2021. **(D)** International collaboration analysis among different countries.

The number of publications and citations in each period can be a direct reflection of the development trend of scientific knowledge in a particular area. After the above-mentioned literature screening, a total of 1,081 publications, including 881 original articles and 200 reviews, were included in the final analysis. The specific distribution of annual publications of ORA research is shown in Figure 2A. As can be seen, despite the appearance of the volatility to decrease at some time points, the annual number of publications related to ORA showed an ascending tendency as a whole and reached its peak in 2019 with a total of 85 documents, which comprised 7.86% of the total quantity. From 1998 to 2019, the average growth rate of scientific publications regarding ORA research was 21.81%. The number of papers in this year, 2021, has reached a count of 75 as of September 14, 2021. When it comes to the number of citations, the cumulative total citations of these publications were 28,473 times (23,692 times after the removal of self-citations), with an average of 26.34 times per publication. As can be seen from the distribution of the annual number of citations (Supplementary Figure 1), it exhibited a linearly increasing trend (*R*^2^ = 0.9508). There were over 2,000 citation frequencies per year in the past 5 years. Collectively, with an in-depth understanding of RA and osteoporosis, the role of osteoporosis in RA has gradually attracted the attention of scholars as reflected from both annual publications and citations quantity. And in recent years, an increasing number of studies in the pathogenesis of osteoporosis in RA have been revealed.

### Basic Knowledge Structures of ORA Field

#### Analysis of Most Productive Countries

A world map depicting the contribution of each country was shown in Figure 2B. According to the indicated color gradient, we can clearly observe that the vast majority of the works of literature were published by researchers from regions such as North America, Western Europe, and Eastern Asia. Comparison of the total number of scientific publications between the three regions showed that authors from Western Europe have published 2.05 times more papers than Eastern Asian authors, and 2.48 times higher than North American authors. Thus, it could be concluded that Western European countries were the most active regions for the ORA-related research. Although the previous bibliometric studies about RA did not compare the number of documents from the three regions, similar findings have been reported by a bibliometric study on postmenopausal osteoporosis, which found that the number of papers published by Western European authors was about 75% greater than North America authors (24–26).

In specific, as displayed in Table 1, the USA has published the most publications in this domain, with 213 (19.7%) documents, followed by Japan and China, and the remaining countries have published <100 articles. In addition, after adjusting by population size and GDP, Norway both occupied the first position with 8.41 papers per million people and 112.5 papers per trillion GDP. As we all know that Norway is a welfare state, where both primary and specialist health care is provided by well-developed publicly funded services. Statistics have revealed that apart from Luxembourg, there is no country spending more on publicly financed health care per capita than Norway (33). Medical investment from the government may be a key incentive for scientific research output. Moreover, our correlation analysis results showed that there was no significant correlation between the number of publications and demographic data (*r* = 0.404, *p* = 0.077), while publication counts and GDP has a high positive correlation (*r* = 0.852, *p* < 0.001). This outcome further illustrates that economic strength is an important factor affecting scientific output. As for the *h*-index, it is defined as the number of publications for an individual, *h*, each acquiring at least *h* citations. The metric thus enables an assessment of the quality and quantity of publications from a country, author, or journal. In this study, the USA (34), UK (35), Netherlands (33), Germany (28), and Italy (28) were the top five countries with the highest *h*-index. The value of this metric might be influenced by the time factor, that is, the relatively new entrants in this field have not accumulated sufficient citations. As can be seen from Figure 2C, prior to 2011, the USA, Japan, and the UK dominated in this field in terms of publications counts, while China experienced rapid growth since 2015, and even surpassed the USA for the first time in 2019. This tendency seems consistent with that of the economic growth process in China. Predictably, the *h*-index in China may further increase in the near future.

**Table 1:** Top 20 countries with the most publications related to ORA research.

**Rank**	**Country**	**Contribution**	**% of 1,081**	**Number of papers per trillion GDP**	**Number of papers per million people**	***h*-index**	**ACI**
1	USA	213	19.70	9.94	0.65	46	33.5
2	Japan	145	13.41	28.54	1.15	24	16.54
3	China	109	10.08	7.09	0.08	23	17.11
4	UK	96	8.88	33.92	1.44	36	48.03
5	Germany	72	6.66	18.65	0.87	28	38.21
6	Netherlands	65	6.01	71.43	3.75	32	54.09
7	Italy	63	5.83	31.50	1.04	28	41.86
8	France	62	5.74	22.79	0.92	26	34.15
9	Norway	45	4.16	112.50	8.41	25	47.31
10	Sweden	43	3.98	81.13	4.18	19	32.28
11	South Korea	41	3.79	24.85	0.79	13	22.66
12	Canada	31	2.87	17.82	0.82	18	28.71
13	Denmark	29	2.68	82.86	4.98	19	29.21
14	Australia	28	2.59	20.00	1.10	16	53.32
15	Spain	23	2.13	16.55	0.49	13	34.39
16	Finland	22	2.04	81.48	3.99	14	20.18
17	Turkey	21	1.94	27.63	0.25	11	14.81
18	Austria	20	1.85	44.44	2.25	15	66.6
19	Belgium	20	1.85	37.74	1.74	15	41.65
20	Brazil	17	1.57	9.24	0.08	12	19.59

*Rank, based on the number of total publications; GDP, Gross Domestic Product; ACI, Average Citations per Item*.

*Publications from Taiwan, Hong Kong, and Macau were assigned to China, and those from England, Northern Ireland, Scotland, and Wales were reclassified to the UK. The Demographic and GDP data were downloaded from the official website of the People's Republic of China, and the World Bank official website (https://data.worldbank.org.cn/)*.

Additionally, ACI is another indicator that reflects the value of the paper and its contribution to science. It is evident from Table 1 that China and Japan have occupied the second and third positions with regard to the number of publications, but the ACI was much lower than that of some European and American countries. As a result, except for quantity increase, there is still a need for improving the quality of publications. Figure 2D displays the international cooperation among different countries. The line thickness between the two countries indicates the strength of cooperation. It can be seen that the USA collaborated most closely with China, Japan, the UK, and Italy. Overall, most of the collaborative relationships are mainly confined to European, American, and East Asian countries. Cooperation in less developed nations needs to be further enhanced.

#### Analysis of the Most Prolific Institutions

**Figure 3:**
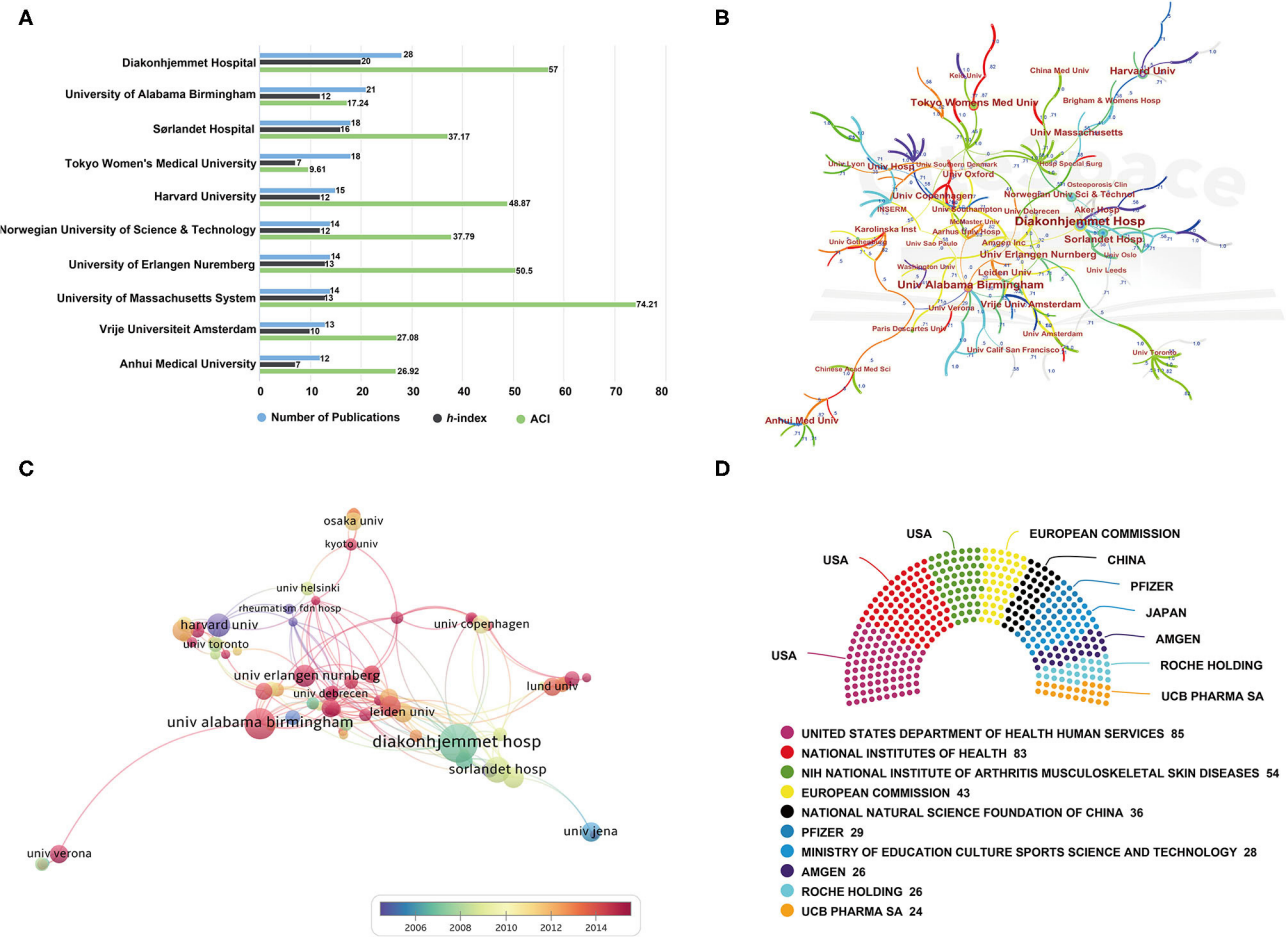
**(A)** The publication counts, *h*-index, and ACI of the top 10 most prolific institutions. **(B)** Visualization map of institution cooperation generated by CiteSpace software. **(C)** Overlay visualization map of institution co-authorship analysis generated by VOSviewer software. **(D)** The top 10 most active funding agencies in ORA research.

As for the analysis of institutions, it was roughly estimated that more than 1,500 institutions have made contributions to this field. The bar graph of Figure 3A demonstrated the publication counts, *h*-index, and ACI of the top 10 most prolific institutions in detail. Of these, three are from the USA, three are from Norway, and the remaining four are from Japan, Germany, Netherlands, and China. To be specific, Diakonhjemmet Hospital in Norway ranked first with 28 articles. The University of Alabama Birmingham from the USA was in second place with 21 publications, while Sørlandet Hospital from Norway and Tokyo Women's Medical University from Japan tied for third place with 18 publications. In terms of other quantitative indices, like *h*-index and ACI, Diakonhjemmet Hospital has the highest *h*-index of 20, and Sørlandet Hospital followed suit (16). The top three institutions with the highest value of ACI were the University of Massachusetts System (74.21 times), Diakonhjemmet Hospital (57 times), and the University of Erlangen Nuremberg (50.5 times). It is interesting to notice that despite the publication counts and *h*-index being not high, the ACI in the University of Massachusetts System was much higher than other institutions. One possible reason is that several studies from this institution have attracted enormous attention. We observed that a multicenter study including participants from the University of Massachusetts System, which explored the prevalence of comorbidities in RA has received more than 406 citations (36). Due to this, as mentioned in previous studies, citations or ACI may not fully capture the impact of scientific work, and evaluate the impact of an individual or institution (37).

Additionally, in our increasingly interdependent and globalized world, it is generally accepted that cross-country and inter-organizational collaboration is important ways to improve research quality and productivity. In this study, institution cooperation analysis was also conducted by CiteSpace software. As seen in Figure 3B, collaborations between institutions are scattered within high-income countries such as North America and Western European countries. Despite the fact that some Asian countries have made great contributions in the case of publication counts, institutions in these regions do not form a cooperation network, indicating a lack of academic exchange among Asian countries as well as research institutions. In addition, of all these institutions, the University of Alabama Birmingham had the highest centrality, with a betweenness centrality (BC) value of 0.13. BC is an indicator of the centrality of a node, which can reflect the importance of nodes within the networks. Generally, nodes with a BC value of more than 0.1 occupy pivotal positions connecting a large number of nodes and are usually identified as hubs of nodes displayed in purple rings (21, 30). It can be seen that the University of Alabama Birmingham was the only institution with a BC value of more than 0.1, which suggests that other institutions have not formed a strong influence in the field. Therefore, as pointed out by other researchers, there is an urgent need to remove academic barriers, improve cooperation and communication in different research institutions and teams (35, 38).

Apart from CiteSpace software, we also performed a co-authorship analysis of institutions by VOSviewer. Co-authorship analysis is a commonly used method to establish similarity relationships among individuals or groups through the number of co-authored publications (28, 39). As illustrated in the overlay visualization map in Figure 3C, nodes that represent institutions were marked by different colors based on the average appearing year (AAY) of each institution. According to the color gradient indicated in the lower right corner, it could be found that several institutions, e.g., Harvard University, Tampere University Hospital, and University of California San Francisco, were given purple color with the smaller values of AAY, suggesting that most of the researchers in these institutions were the relatively earlier entrants in this field. By contrast, many institutions marked with red or dark red color could be the relatively new participants of ORA research.

#### Analysis of Most Active Funding Agencies

As noted above, the economic foundation plays an important role in scientific development. In view of this, a brief summary of the top 10 most active funding agencies and sponsors in this area is provided in Figure 3D. In the case of distribution, funding organizations from the USA including the United States Department of Health Human Services, National Institutes of Health, and NIH National Institute of Arthritis Musculoskeletal Skin Diseases, occupied the top three positions contributed to ORA research, with 85, 83, and 54 studies, respectively. The remaining were from European Union, China, and Japan, and some pharmaceutical companies such as Pfizer and Amgen, among others. As is evident from these results, in addition to the well-established institutions, the USA maintained its leading position in the domain of ORA research cannot be separated from the support of adequate funding.

#### Analysis of the Most Influential Authors

**Figure 4:**
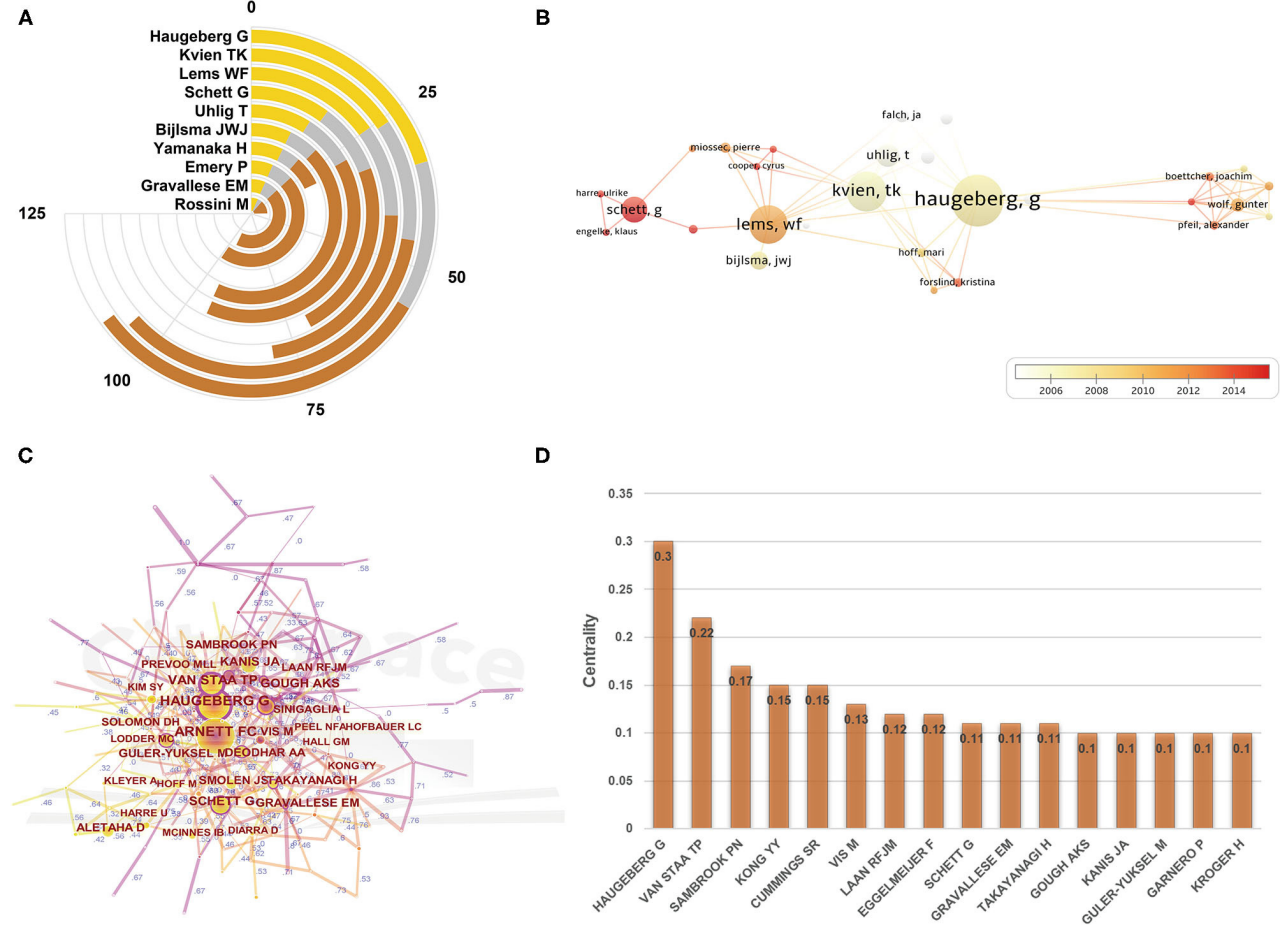
**(A)** The publication counts, *h*-index, and ACI of the top 10 most prolific authors. **(B)** Overlay visualization map of author co-authorship analysis generated by VOSviewer software. **(C)** Visualization map of author co-citation analysis by using CiteSpace software. **(D)** All the authors with a centrality value of more than 0.1 (author co-citation analysis).

The number of scientific publications written by one author is able to represent the degree of research activity and contribution in the field. More than 5,000 authors participated in the publication of these 1,081 documents. From the perspective of publication counts (Figure 4A), Haugeberg G from Norway was the author with the highest number of publications, followed by Kvien TK, and Lems WF. Apart from that, they were also the top three authors with the highest *h*-index. Of them, Haugeberg G and Kvien TK came from the same research institution. In the year 2000, they published work about bone mineral density (BMD) and frequency of osteoporosis in women RA patients aged 20–70 years. The results of their study reported that a 2-fold increase in osteoporosis was found in this population (4). This report has risen much attention in the field of ORA research and has been cited over 350 times up to now. In addition, Haugeberg and colleagues have also completed several population-based cohort studies that evaluated the impact of drug interventions including prednisolone, and infliximab combined with methotrexate on the bone density of RA patients. The results revealed that RA-related bone loss in hand bone can be decelerated by prednisolone (40). In the meantime, they also found that infliximab was potent enough to arrest inflammatory-related loss of hip bone density in early RA patients (41).

Scholars devoted to different research priorities have unique professional knowledge, among which cross-cooperation can promote communication and productivity of a certain research subject. In addition, an analysis of the co-authorship of authors is advantageous for researchers to learn existing partnerships and develop potential cooperative subjects. In Figure 4B, an overlay visualization map of author co-authorship analysis was generated by VOSviewer software. As can be seen that several research clusters were created, and each cluster was radiated by one or two core authors such as Haugeberg G, Kvien TK, Lems WF, and Schett G. Overall, there were only a few links between different clusters, indicating that the communication and collaboration in this domain have not been well developed. Besides, according to the color gradient indicated in the lower right corner, one can also understand the AAY of each author, that marked with different colors. As it can be noticed, the cluster centered on Schett G seems to be relatively younger researchers in this field.

The co-citation relationship refers to two authors/works of literature appearing together in the reference list of a third document (28). The author co-citation analysis is often used to reveal the key authors in a co-citation network of a particular field. Generally, frequently cited authors are thought to have a greater influence than those less cited. And authors who are jointly cited are likely to focus on similar research areas. As displayed in Figures 4C,D, Haugeberg G had the largest BC value (0.3), ranked first among the top 10 co-cited authors, followed by Van Staa TP (0.22) and Sambrook PN (0.17). Professor Van Staa TP works at the University of Southampton. He and co-workers published a study estimating the long-term absolute fracture risk of RA patients (9). The study found that patients with RA were at increased risk of osteoporotic fractures, and they suggested this could be due to the use of oral glucocorticoids, which seems to be inconsistent with some previous studies (40). As for professor Sambrook PN from Garvan Institute of Medical Research, he and colleagues primarily devoted to the potential pathogenetic mechanisms that cause generalized osteoporosis in RA (42, 43). From the above results, it is clear that Haugeberg G was the most influential author in the ORA field, either from the volume of publications or the co-authorship and co-citation perspective.

#### Analysis of the Higher-Impact Journals

For centuries, scientific publications have always been essential tools for science communication of scientists and researchers in all fields. The presentation of research results in an international peer-reviewed journal is an essential component to establish effective scientific communication (23, 28). The analysis of the distribution of journal sources is helpful for researchers to quickly find the most appropriate journals for their articles. With preliminary statistics, all these publications related to ORA research were distributed in more than 1,000 journals, and Table 2 summarized the basic information on the top 20 most prolific journals. Of these, *Journal of Rheumatology* (64, 5.92%) had the highest number of outputs, followed by *Rheumatology* (47, 4.35%), *Clinical Rheumatology* (44, 4.07%), *Osteoporosis International* (44, 4.07%), and *Rheumatology International* (44, 4.07%). Furthermore, the JIF of a journal is an important factor parameter to evaluate its value and that of included publications. This concept of the JIF of the journal developed by Garfield (44), was intended to be a measurement of a 2-year moving average citation of a journal. Among the top 20 academic journals, *Annals of the Rheumatic Diseases* (19.103) has the highest JIF, followed by *Arthritis & Rheumatology* (10.995), and *Frontiers in Immunology* (7.561). Journal Citation Reports also split journals belonging to the same WoS categories into four equal parts based on JIF value, among which the top 25% attributed to Q1 and the top 25–50% being Q2, and so forth. It can be seen from Table 2 that 20% of journals belong to Q1. As for the research domain of these journals, 70% of them were classified into rheumatology.

**Table 2:** Top 20 journals with most publications in the field of ORA research.

**Ranking**	**Sources title**	**Output**	**% of 1,081**	**JIF (2020)**	**Quartile in category (2020)**
1	*Journal of Rheumatology*	64	5.92	4.666	Q2
2	*Rheumatology*	47	4.35	7.58	Q1
3	*Clinical Rheumatology*	44	4.07	2.98	Q3
4	*Osteoporosis International*	44	4.07	4.507	Q2
5	*Rheumatology International*	44	4.07	2.631	Q4
6	*Annals of the Rheumatic Diseases*	43	3.98	19.103	Q1
7	*Arthritis Research Therapy*	35	3.24	5.156	Q2
8	*Clinical and Experimental Rheumatology*	32	2.96	4.473	Q2
9	*BMC Musculoskeletal Disorders*	25	2.31	2.362	Q2/Q4
10	*Modern Rheumatology*	23	2.13	3.023	Q3
11	*Arthritis and Rheumatology*	24	2.22	10.995	Q1
12	*Calcified Tissue International*	21	1.94	4.333	Q2
13	*Journal Of Bone and Mineral Metabolism*	20	1.85	2.626	Q3/Q4
14	*Best Practice Research in Clinical Rheumatology*	15	1.39	4.098	Q3
15	*Bone*	15	1.39	4.398	Q2
16	*Joint Bone Spine*	15	1.39	4.929	Q2
17	*Scandinavian Journal of Rheumatology*	14	1.30	3.641	Q3
18	*Arthritis Care Research*	13	1.20	4.794	Q2
19	*Current Opinion in Rheumatology*	13	1.20	5.006	Q2
20	*Frontiers in Immunology*	12	1.11	7.561	Q1

*After checking the names of the journals, we ascertained that Arthritis and Rheumatism changed their name to Arthritis and Rheumatology. This was also found in the Journal of Bone and Joint Surgery Br and Bone and Joint Journal. The data from the same journals were merged*.

**Figure 5:**
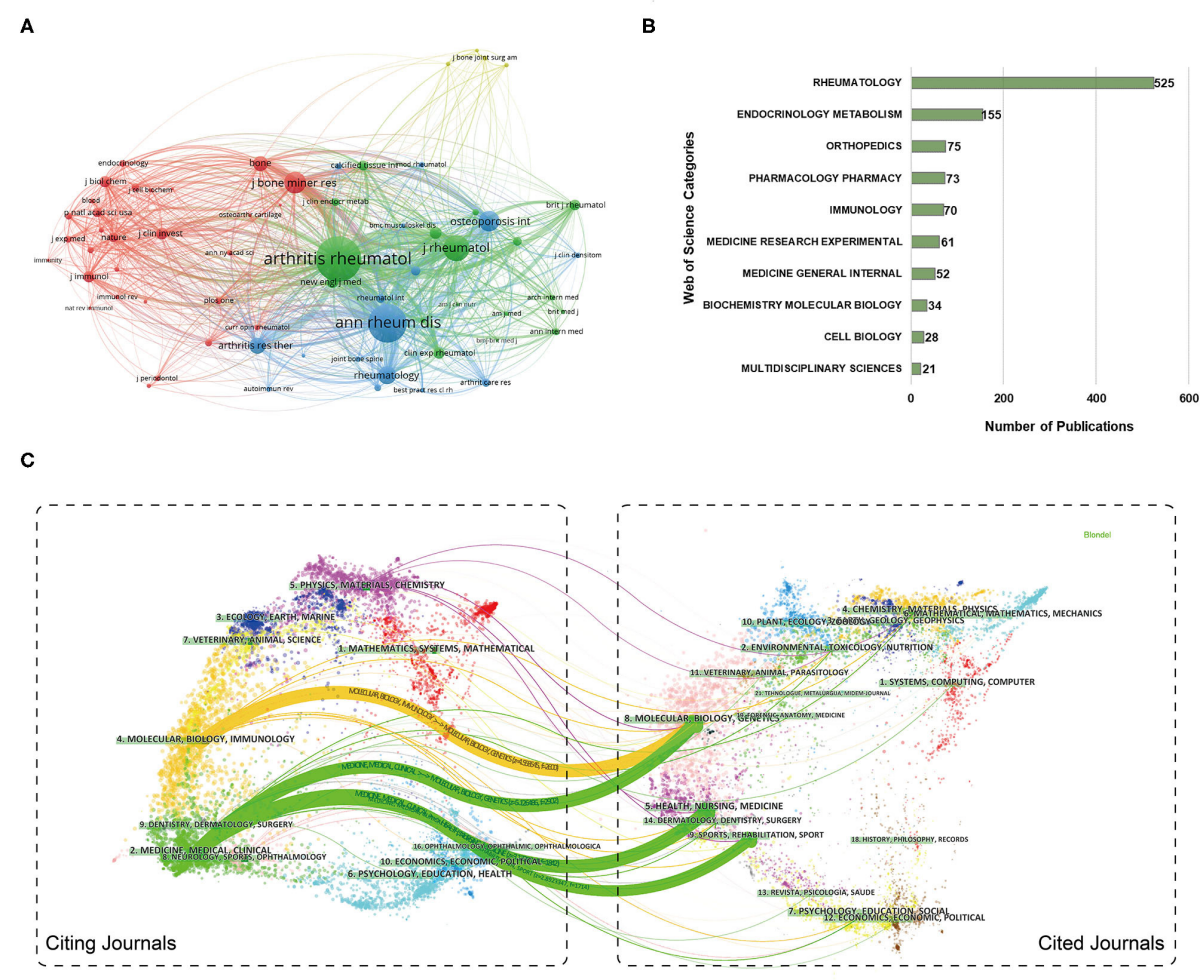
**(A)** Network visualization map of journal co-citation analysis based on VOSviewer software. **(B)** The top 10 most relevant WoS subject categories. **(C)** The dual-map overlay of academic journals in the field of ORA research (generated by CiteSpace software).

In terms of the publisher, of these top 20 journals, eight were from England, six were from the USA, and the others came from Canada, Germany, Italy, Japan, France, and Switzerland. Remarkably, the majority of these active journals were based in Western Europe and North America. Although the East Asiatic region was also one of the predominant contributors in this field, there was only one publisher from Japan, and even no one Chinese journal, indicating that Asian countries, especially China should strengthen the development of international journals to further improve the academic influence in the field. It is laudable that the Chinese government has invested a large number of resources into international journals construction, and multiple incentives have been promulgated in recent years (45). With the exception of publication counts, the influence of a journal also depends on the number of times they are co-cited in a particular research field. In this work, co-citation analysis of journals was performed by using the VOSviewer software. As shown in Figure 5A, journals with a minimum of 100 citations were included in the visualization analysis. There were 66 nodes, four clusters, and 2,144 links in the network map. The top five journals with the largest citations were *Arthritis & Rheumatology, Annals of the Rheumatic Diseases, Journal of Rheumatology, Journal of Bone and Mineral Research*, and *Osteoporosis International*. The results indicated that these journals have published numerous high-profile studies that attracted great attention from researchers interested in this field. Of these, *Arthritis & Rheumatology, Annals of the Rheumatic Diseases*, and *Journal of Rheumatology* mainly focus on studies of the rheumatic diseases, while *Journal of Bone and Mineral Research*, and *Osteoporosis International* are the primary journal containing bone and metabolism-related studies. It is therefore predictable that there may be more high-quality research published in these journals.

#### Analysis of the Most Relevant Subject Categories

In the WoSCC database, each article was labeled with one or more subject categories to facilitate rapid search. The top 10 subject categories in terms of the number of publications were shown in Figure 5B. Consistent with the distribution of journals, Rheumatology, Endocrinology, and Metabolism, Orthopedics was the most predominant subject category that received the most attention in this field. In addition, the dual-map overlay of journals stood for discipline distribution of journals involving ORA research. In the dual-map overlays, the base map for citations was generated from 10,000 journals indexed in WoS (46). After the dataset was entered, the citing trajectories were built in the dual-map overlay module. With this approach, we can see clearly how knowledge flows in different disciplines and identify the hotspot of each discipline. As presented in Figure 5C, the citing journals were on the left, the cited journals were on the right, and colored paths represented the citation relationship. There were following four core citation paths shown in the map. The orange paths indicate that documents published in Molecular/Biology/Immunology journals usually cited documents published in journals belonging to Molecular/Biology/Genetics. The green paths imply that the majority of papers published in the journals of Medicine/Medical/Clinical are likely to be biased to cite papers published in journals within Molecular/Biology/Genetics, Health/Nursing/Medicine, and Sports/Rehabilitation/Sport.

### An Overview of Research Hotspots and Frontiers

#### Analysis of Highly-Cited Studies

The analysis of citations is one of the key methodologies in a bibliometric study. Although there remains some controversy on the value of citation rates (47), it is generally agreed that the number of citations could reflect the impact extent of a publication, and the higher citations frequency indicates a higher academic level in a field (27). Table 3 listed the top 20 most cited papers on ORA. All these studies were published between 2000 and 2016, and 50% of them acquired more than 200 citation times. The majority of studies were published in rheumatology journals such as *Arthritis and Rheumatism, Annals of the Rheumatic Diseases*, and *Rheumatology*. Among them, 12 were original articles and eight were systematic reviews. Specifically, a review entitled “Understanding the dynamics: pathways involved in the pathogenesis of rheumatoid arthritis” published in *Rheumatology* has been cited 420 times and is the top-cited paper in the field (13). This review made a detailed overview of various immune modulators including cytokines and effector cells, and signaling pathways are involved in the pathophysiology of RA. It also summarized the role of cytokines especially IL-6 in the osteoporotic manifestation of RA. The second and third highest cited papers were published by Dougados et al. (36) and Haugeberg et al. (4), which have been discussed in detail previously. In summary, topics of top 20 publications mainly include reviews regarding cytokines and the impact on bone homeostasis (13, 34, 48, 49), epidemiological and clinical assessment of ORA (4, 5, 9, 36, 50), the molecular mechanism underlying RA-associated bone loss (51–53), and pharmacologic intervention studies on ORA (54, 55).

**Table 3:** The top 20 most cited works of literature on ORA.

**Title**	**Journal**	**First Author**	**Publication year**	**Total citations**
Understanding the dynamics: pathways involved in the pathogenesis of rheumatoid arthritis	*Rheumatology*	Choy E	2012	420
Prevalence of comorbidities in rheumatoid arthritis and evaluation of their monitoring: results of an international, cross-sectional study (COMORA)	*Annals of the Rheumatic Diseases*	Dougados M	2014	406
Bone mineral density and frequency of osteoporosis in female patients with rheumatoid arthritis-Results from 394 patients in the Oslo County Rheumatoid Arthritis Register	*Arthritis and Rheumatism*	Haugeberg G	2000	363
Clinical assessment of the long-term risk of fracture in patients with rheumatoid arthritis	*Arthritis and Rheumatism*	van Staa TP	2006	359
Low-dose prednisone therapy for patients with early active rheumatoid arthritis: Clinical efficacy, disease-modifying properties, and side effects-A randomized, double-blind, placebo-controlled clinical trial	*Annals of Internal Medicine*	van Everdingen AA	2002	341
Involvement of receptor activator of NF kappa B ligand and tumor necrosis factor-alpha in bone destruction in rheumatoid arthritis	*Bone*	Romas E	2002	318
Biology of the RANKL-RANK-OPG system in immunity, bone, and beyond	*Frontiers in Immunology*	Walsh MC	2014	314
RANK/RANKL: Regulators of Immune Responses and Bone Physiology	*Annals of the New York Academy of Sciences*	Leibbrandt A	2008	280
Relationship between rheumatoid arthritis and periodontitis	*Journal of Periodontology*	Mercado FB	2001	255
Low-dose prednisolone in addition to the initial disease-modifying antirheumatic drug in patients with early active rheumatoid arthritis reduces joint destruction and increases the remission rate - A two-year randomized trial	*Arthritis and Rheumatism*	Svensson B	2005	251
IL-7 induces bone loss *in vivo* by induction of receptor activator of nuclear factor kappa B ligand and tumor necrosis factor a from T cells	*Proceedings of the National Academy of Sciences of the United States of America*	Toraldo G	2003	209
1,25-Dihydroxyvitamin D-3 Modulates Th17 Polarization and Interleukin-22 Expression by Memory T Cells From Patients With Early Rheumatoid Arthritis	*Arthritis and Rheumatism*	Colin EM	2010	204
Osteoblast physiology in normal and pathological conditions	*Cell and Tissue Research*	Neve A	2011	201
Bone loss before the clinical onset of rheumatoid arthritis in subjects with anticitrullinated protein antibodies	*Annals of the Rheumatic Diseases*	Kleyer A	2014	192
Identification of a novel chemokine-dependent molecular mechanism underlying rheumatoid arthritis-associated autoantibody-mediated bone loss	*Annals of the Rheumatic Diseases*	Krishnamurthy A	2016	184
Classical and paradoxical effects of TNF-alpha on bone homeostasis	*Frontiers in Immunology*	Osta B	2014	183
Bone remodeling in rheumatic disease: a question of balance	*Immunological Reviews*	Walsh NC	2010	166
Evaluation of bone mineral density, bone metabolism, osteoprotegerin and receptor activator of the NF kappa B ligand serum levels during treatment with infliximab in patients with rheumatoid arthritis	*Annals of the Rheumatic Diseases*	Vis M	2006	164
Therapeutic targets in rheumatoid arthritis: the interleukin-6 receptor	*Rheumatology*	Dayer JM	2010	159
A multicenter cross sectional study on bone mineral density in rheumatoid arthritis	*Journal of Rheumatology*	Sinigaglia L	2000	155

#### References Co-citation Analysis

**Figure 6:**
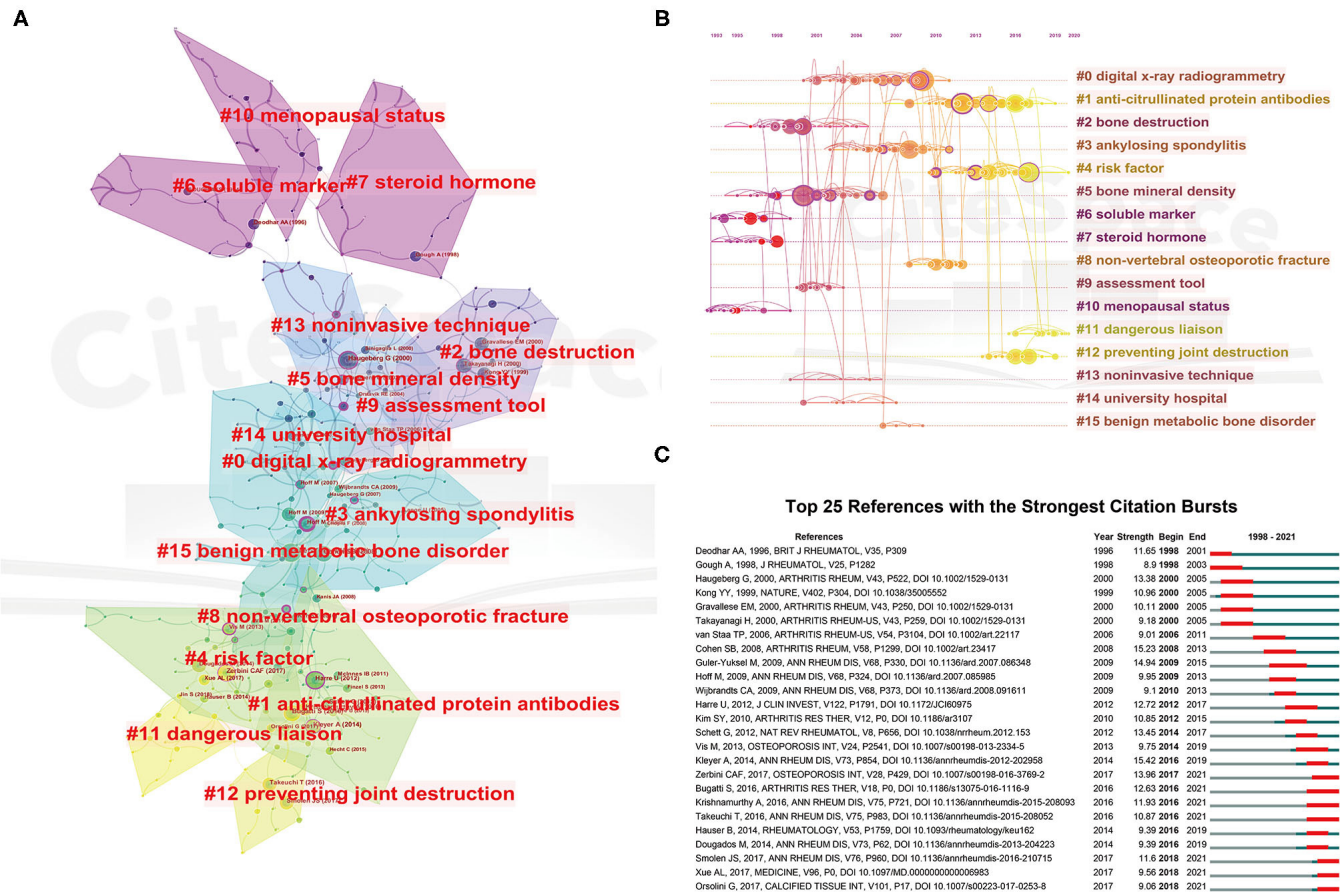
The cluster view map **(A)** and timeline view map **(B)** of reference co-citation analysis were generated by CiteSpace. **(C)** Visualization map of top 25 references with the strongest citation bursts involved in ORA.

Furthermore, reference co-citation analysis was a valuable technique to assess the evolution and trace the developmental frontiers of any research field (21). After running the bibliometric analysis in CiteSpace, a visualization network of cited references was plotted in Figure 6A. By using the clustering function, the whole network map could be divided into several different clusters, which studies within the same cluster might have similar research topics than studies from other clusters. It should be noted that the modularity value (Q-value) and mean silhouette value (S-value) are two important parameters to evaluate the significance of community structure, that is, a Q > 0.3 and S > 0.7 corresponds to a significant clustering (56). In this study, the Q value was 0.8097, indicating the reasonableness of this network. The mean S-value was 0.9418, in which all the S-values of clusters #0–#15 were larger than 0.88, suggesting the good homogeneity of these clusters. As can be seen from Figure 6A and Table 4, “digital X-ray radiogrammetry” was the largest cluster (#0), followed by “anti-citrullinated protein antibodies” (#1), and “Bone destruction” (#2). Along with this, we also provided the timeline view for the major clusters in Figure 6B, from which the evolution characteristics of each cluster could be told at a glance based on the time axis or the average year of the clusters in Table 4. One can see that the research focus has shifted from “soluble marker” (#6), and “menopausal status” (#10), and “steroid hormone” (#7) to “anti-citrullinated protein antibodies” (#1), “risk factor” (#4), “dangerous liaison” (#11), and “preventing joint destruction” (#12).

**Table 4:** The clusters information of co-cited references.

**Cluster**	**Size**	**Silhouette**	**Label**	**Mean**
**ID**				**(Year)**
#0	41	0.919	Digital X-ray radiogrammetry	2005
#1	40	0.955	Anti-citrullinated protein antibodies	2013
#2	38	0.927	Bone destruction	2000
#3	37	0.926	Ankylosing spondylitis	2006
#4	36	0.881	Risk factor	2013
#5	33	0.912	Bone mineral density	2000
#6	25	0.989	Soluble marker	1994
#7	23	0.965	Steroid hormone	1995
#8	23	0.952	Non-vertebral osteoporotic fracture	2010
#9	22	0.943	Assessment tool	2001
#10	22	1	Menopausal status	1994
#11	16	0.952	Dangerous liaison	2018
#12	14	0.949	Preventing joint destruction	2016
#13	10	0.984	Noninvasive technique	2002
#14	8	1	University hospital	2003
#15	6	0.986	Benign metabolic bone disorder	2007

#### Analysis of References With Citation Burst

Moreover, burst detection, an algorithm developed by Kleinberg (57), was an effective analytic tool to capture the sharp increases of references or keywords popularity within a specified period. This function can serve as an efficient way to identify concepts or topics that were actively discussed during some period of time. In the present study, the burst detection algorithm was applied to extract key references and keywords for ORA research. Figure 6C illustrated the top 25 references with the strongest citation bursts. In this map, the blue lines indicated the time interval, and the red part represented the time period when the reference burst occurred. Among these references, the reference with the strongest burst value was written by Kleyer et al. (52). In this study, they found that structural bone damage had already begun before the clinical onset of arthritis in anticitrullinated protein antibodies (ACPA) positive individuals. This finding corrected the previous concept that bone destruction was an exclusive consequence of synovitis in RA patients. In addition, as also can be seen that the first burst of co-cited reference began in 1998 due to a review summarizing bone mass measurement especially of the hand in RA patients, and the burst lasted for 4 years (58). Notably, although the burst in the majority of references was over, the burst in several references is still ongoing, indicating that these research topics are being of continuous concern in recent years. Of these, most of these references involved ACPA and therapies preventing joint destruction. For instance, one phase II clinical trial evaluated the efficacy and safety of denosumab on RA patients with risk factors of joint destruction (59). The results indicated that denosumab was able to significantly inhibit the progression of bone erosion at 12 months in comparison with the placebo group. Zerbini et al. (60) summarized the evidence of biological therapies on BMD, bone turnover markers, and fragility fractures in RA patients. In terms of studies related to ACPA, Orsolini et al. (61) analyzed the effect of ACPA on systemic BMD in established RA patients. The multivariate analysis confirmed the negative effect of ACPA positive on BMD of femoral sites, but not at the lumbar spine. A similar result was also reported by Bugatti and colleagues (62). They suggested that systemic reduced BMD in patients with early RA was associated with ACPA positivity and high rheumatoid factor levels. While Krishnamurthy and collaborators further dissected the role of ACPAs in osteoclast in osteoclast activation and identified the key cellular mediators of this process (53). Their findings suggested that IL8-dependent osteoclast activation could be an early event of initiating joint-specific inflammation in ACPA-positive patients.

#### Analysis of Most Frequently Appearing Keywords

**Figure 7:**
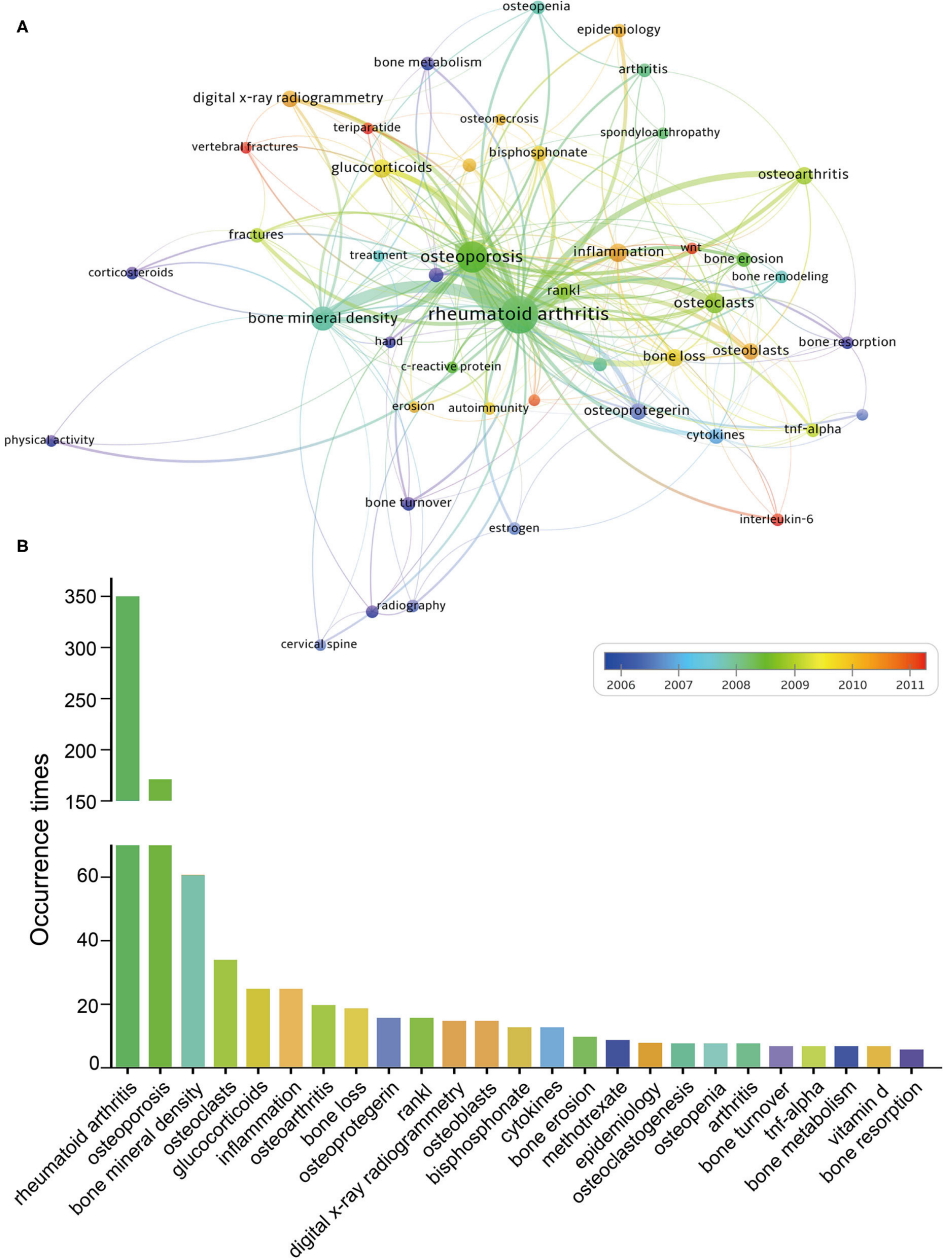
**(A)** Overlay visualization map of keywords co-occurrence analysis. **(B)** The top 25 keywords with the largest occurrence times.

In addition to references, keywords are also representative of the main topics and core content of a specific subject (63). For bibliometrics, another prevalent way to identify hot research topics was keywords co-occurrence analysis. In a co-occurrence analysis, the relatedness of keywords is determined according to the number of documents in which they occur together (28). In this study, author keywords were extracted from 1,081 publications and analyzed by VOSviewer. After excluding several meaningless keywords, and merging keywords with the same meaning, 46 keywords were identified. Figure 7A presented the overlay visualization map of the most frequently used keywords in ORA research. The size of nodes is proportional to the occurrence times of keywords, and the relative distance between two nodes approximates the strength of their relationship. The thicker the lines between two nodes, the higher the frequency of their co-occurrence (28). Figure 7B showed the frequency distribution of the top 25 most common keywords. It can be seen that besides the keywords “rheumatoid arthritis” and “osteoporosis”, the other common keywords mainly focused on the molecular mechanisms and signaling pathways studies on ORA. It is commonly held that high levels of pro-inflammatory cytokines and immune system dysregulation played a critical role in the progression of ORA (1, 64). Thus, theoretically, the drugs that reduce inflammation, especially biological agents, may simultaneously alleviate inflammatory conditions and prevent bone loss (65). Although results are still debated, elucidation of these molecular mechanisms of ORA may facilitate the discovery of novel therapeutic strategies.

In addition, all these keywords were also marked with different colors according to the AAY by VOSviewer. Keywords that appeared relatively earlier were colored in blue, while keywords with a more recent appearance were colored in red. These keywords such as “bone turnover,” “bone metabolism,” “bone resorption,” and “methotrexate” were the major topic during the early stage. While the keywords “teriparatide” (66), “interleukin-6” (67), “Wnt” (68), and “vertebral fractures” (69) showed a relatively latest AAY, which indicated that this topic may have gained increasing attention recently, and have the potential to become research hotspots in the near future. Take teriparatide as an example, it is an osteoanabolic agent that significantly increases cortical and trabecular bone microstructure indices and is also the only available anabolic agent for osteoporosis in many countries (70). Multiple previous studies have revealed that daily teriparatide treatment was able to significantly increase the BMD and bone strength of the lumbar spine and reduce the rates of clinical fractures (66, 71). However, results from large-sample randomized controlled studies are currently lacking, which could be the future direction of next research.

## Limitation

The present study had some limitations inherent in bibliometrics as well. Firstly, the dataset consisted of only data from the WoSCC database but neglected the other large databases, which could miss a few related studies. However, as described in previous studies, WoSCC was the most commonly applied database for bibliometric analysis (17, 28, 39). And data from WoSCC was large enough to reflect the current state of ORA research. Moreover, different databases are characterized by different features including output formats of files and count of citations. Merging of the databases may not optimal choice. Secondly, we only selected studies published in the English language and ignored non-English language publications, which means that the contributions of non-English speaking countries are likely to be underestimated. Thirdly, since the WoSCC database is continually updated, the influence of recently published high-quality articles may also be underestimated as they may not accumulate sufficient citations.

## Conclusion

To our knowledge, this was the first-ever study to conduct a comprehensive bibliometric analysis of publications related to ORA from 1998 to 2021. Our findings demonstrates that the role of osteoporosis in RA has gradually attracted the attention of scholars as reflected in both annual publications and citations quantity. So far, the United States has been the leader in this field, which cannot be separated from sufficient funding sources. Diakonhjemmet Hospital and professor Haugeberg G were the most prolific institution and influential authors, respectively. *Journal of Rheumatology* and *Arthritis & Rheumatology* were the most productive and influential journals in ORA research, with the largest number of publications and citations, respectively. According to the burst references, “anti-citrullinated protein antibodies” and “preventing joint destruction” have been recognized as the hot research issues in the domain. Besides that, a keywords co-occurrence analysis identified “teriparatide,” “interleukin-6,” “Wnt,” and “vertebral fractures” as important future research directions, which deserves further attention. All in all, researchers especially new entrants could benefit from this bibliometric analysis as they could clearly understand the fundamental knowledge structure including countries, institutions, authors, and journals in this field, and be inspired by the analysis of research hotspots and frontiers. In addition, this study could also provide a valuable reference for policymakers and funders to make more correct investments.

## Data Availability Statement

The original contributions presented in the study are included in the article/Supplementary Material, further inquiries can be directed to the corresponding author.

## Author Contributions

HW, KC, and ZS designed the study. HW, QG, LT, and YW collected the data. HW, KC, YW, LT, and ZS analyzed the data and drafted the manuscript. HW, QG, and ZS revised and approved the final version of the manuscript. All authors have read and approved the submitted version.

## Funding

This work was supported by the Tianjin Municipal Health Bureau (Grant Number: 14KG115) and the Key Program of the Natural Science Foundation of Tianjin (Grant Number: 20JCZDJC00730).

## Conflict of Interest

The authors declare that the research was conducted in the absence of any commercial or financial relationships that could be construed as a potential conflict of interest.

## Publisher's Note

All claims expressed in this article are solely those of the authors and do not necessarily represent those of their affiliated organizations, or those of the publisher, the editors and the reviewers. Any product that may be evaluated in this article, or claim that may be made by its manufacturer, is not guaranteed or endorsed by the publisher.
