# Supplementary material for: Mapping Knowledge Structure and Themes Trends of Osteoporosis in Rheumatoid Arthritis: A Bibliometric Analysis
Source: Front Med (Lausanne). 2021 Nov 23;8:787228. doi: 10.3389/fmed.2021.787228 (PMC8650090; doi:10.3389/fmed.2021.787228)
Supplement: Supplementary file 1 [file Data_Sheet_1.DOCX]

Supplementary Material

# Supplementary Figures and Tables

## Supplementary Figure


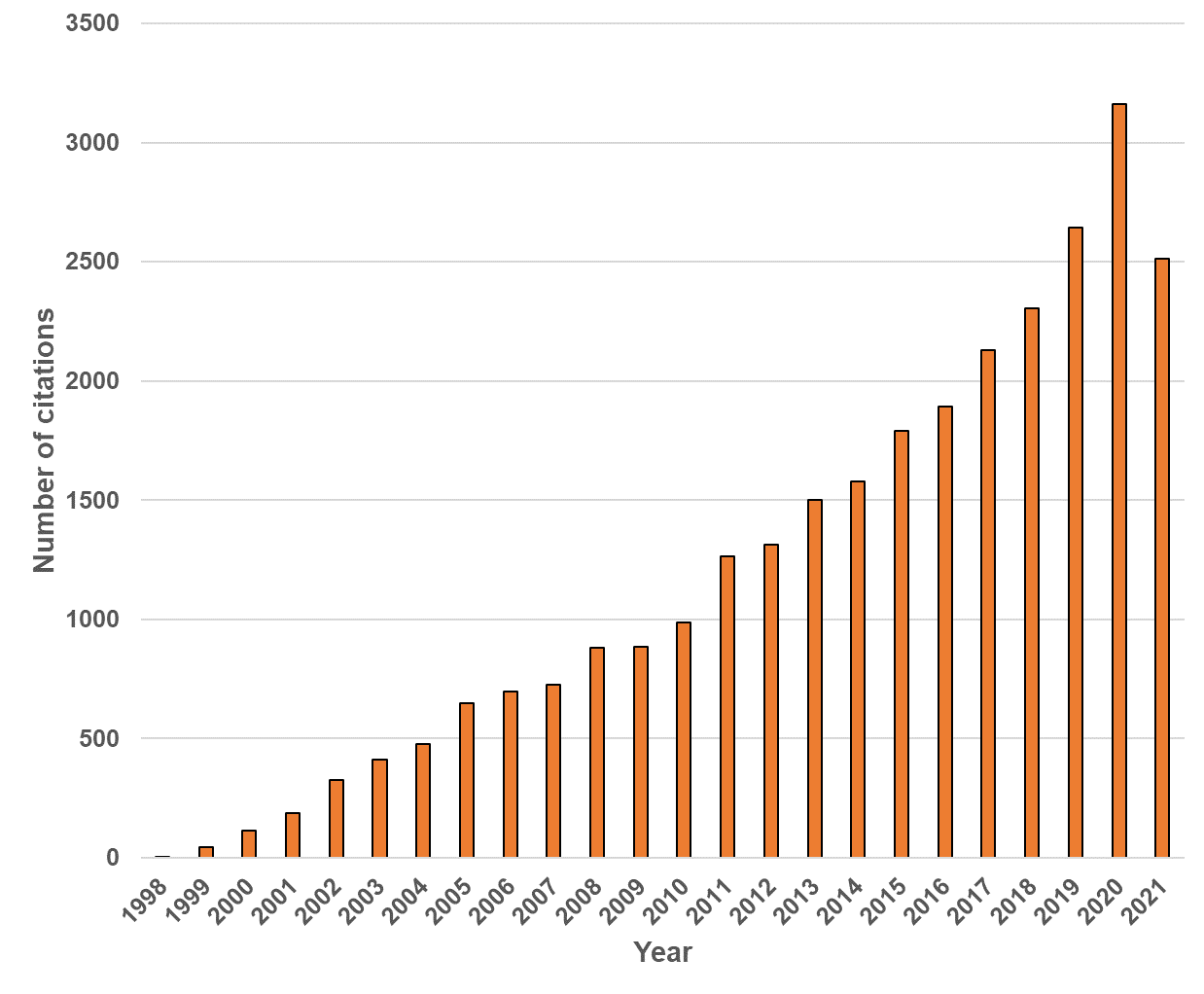


**Supplementary Figure 1.** Distribution of the annual number of citations regarding osteoporosis in RA research from 1998 to 2021.
